# Mismatch repair protein MSH2 regulates translesion DNA synthesis following exposure of cells to UV radiation

**DOI:** 10.1093/nar/gkt793

**Published:** 2013-09-12

**Authors:** Lingna Lv, Fengli Wang, Xiaolu Ma, Yeran Yang, Zhifeng Wang, Hongmei Liu, Xiaoling Li, Zhenbo Liu, Ting Zhang, Min Huang, Errol C. Friedberg, Tie-Shan Tang, Caixia Guo

**Affiliations:** ^1^Laboratory of Cancer Genomics and Individualized Medicine, Beijing Institute of Genomics, Chinese Academy of Sciences, Beijing 100101, China, ^2^State Key Laboratory of Biomembrane and Membrane Biotechnology, Institute of Zoology, Chinese Academy of Sciences, Beijing 100101, China and ^3^Department of Pathology, University of Texas Southwestern Medical Center, Dallas, TX 75390, USA

## Abstract

Translesion DNA synthesis (TLS) can use specialized DNA polymerases to insert and/or extend nucleotides across lesions, thereby limiting stalled replication fork collapse and the potential for cell death. Recent studies have shown that monoubiquitinated proliferating cell nuclear antigen (PCNA) plays an important role in recruitment of Y-family TLS polymerases to stalled replication forks after DNA damage treatment. To explore the possible roles of other factors that regulate the ultraviolet (UV)-induced assembly of specialized DNA polymerases at arrested replication forks, we performed immunoprecipitation experiments combined with mass spectrometry and established that DNA polymerase kappa (Polκ) can partner with MSH2, an important mismatch repair protein associated with hereditary non-polyposis colorectal cancer. We found that depletion of MSH2 impairs PCNA monoubiquitination and the formation of foci containing Polκ and other TLS polymerases after UV irradiation of cells. Interestingly, expression of MSH2 in Rad18-deficient cells increased UV-induced Polκ and REV1 focus formation without detectable changes in PCNA monoubiquitination, indicating that MSH2 can regulate post-UV focus formation by specialized DNA polymerases in both PCNA monoubiquitination-dependent and -independent fashions. Moreover, we observed that MSH2 can facilitate TLS across cyclobutane pyrimidine dimers photoproducts in living cells, presenting a novel role of MSH2 in post-UV cellular responses.

## INTRODUCTION

Translesion DNA synthesis (TLS) is a mode of DNA damage tolerance that uses specialized DNA polymerases to support DNA synthesis past a spectrum of template strand base damage, thereby preventing stalled replication forks from collapse and possible cell death (1). Ten different specialized DNA polymerases in mammalian cells have been shown to support TLS *in vitro*. These enzymes are devoid of 3′→5′ proofreading exonuclease activity and replicate undamaged DNA *in vitro* with low fidelity and weak processivity (2). Among them, DNA polymerases kappa (Polκ), iota (Polι), eta (Polη) and REV1 belong to a novel DNA polymerase family (the Y-family) (3,4).

Each of the Y-family polymerases exhibits a preference for the replicative bypass of specific types of base damage in DNA. For example, Polκ and Polη support accurate bypass of benzo[*a*]pyrene diol epoxide guanine adducts (B[a]P-dG) and solar ultraviolet (UV) radiation-induced cis–syn thymine–thymine cyclobutane–pyrimidine dimers (CPDs), respectively (2,5,6). Polη-deficient cells manifest UV radiation-induced mutagenesis, and a markedly elevated predisposition to UV radiation-induced skin cancer has been observed in Polη-deficient humans and mice (3,4,7). Similarly, Polκ-deficient mouse embryonic fibroblasts and embryonic stem cells are sensitive to the killing effects of benzo[*a*]pyrene, and exhibit enhanced benzo[*a*]pyrene diol epoxide-induced mutagenesis (8–10). Additionally, increased spontaneous mutagenesis has been observed in some tissues from Polκ-deficient mice (9), suggesting that some, if not all TLS polymerases, are required for the maintenance of genome stability.

These observations notwithstanding, over-expression of some specialized DNA polymerases results in elevated genomic instability that manifests with enhanced mutation rates as well as DNA strand breaks (3,11,12). Given the low fidelity of TLS polymerases copying undamaged templates *in vitro*, it is believed that TLS processes *in vivo* are strictly regulated to keep TLS polymerases mainly functioning at their cognate substrates in an error-free fashion. Consistent with these observations, dysregulation of Polκ recruitment to replication forks promotes genomic instability (13).

TLS in mammalian cells is promoted by monoubiquitination of proliferating cell nuclear antigen (PCNA). A number of studies have shown that monoubiquitinated PCNA exhibits enhanced interaction with Polη, Polκ, Polι and REV1, relative to unmodified PCNA (14–19). In response to UV radiation, PCNA is monoubiquitinated at Lys164 by the ubiquitin-conjugating enzyme Rad6 and its cognate ubiquitin ligase Rad18 (20,21). The upstream signal that activates PCNA monoubiquitination (PCNA-mUb) *in vivo* is replication protein A (RPA)-coated single-stranded DNA (ssDNA) at sites of stalled forks, in which RPA targets Rad18 to its sites of action (22). Monoubiquitinated PCNA is deubiquitinated primarily by the ubiquitin-specific protease 1 (USP1) (23). More recently, several other cellular constituents have been shown to regulate PCNA-mUb, notably p21 (24), NBS1 (25), C1orf24 (26–30). Other as yet unidentified cellular constituents are conceivably involved in regulating both PCNA-mUb and TLS in normal cells.

Although PCNA-mUb is required for optimal TLS, several lines of evidence indicate the existence of TLS pathways that are independent of PCNA-mUb in mammalian cells (31,32). In this scenario, some if not all specialized DNA polymerases can be recruited to damaged DNA in the absence of PCNA-mUb, and also support TLS, albeit with significantly lower efficiency. The precise mechanism(s) by which specialized DNA polymerases are recruited to damaged sites in the absence of PCNA-mUb is unknown.

In this study, we report that Polκ and REV1 associate physically with the mismatch repair (MMR) protein MSH2. We also show that depletion of MSH2 reduces Polκ and REV1 focus formation, the levels of PCNA-mUb and the bypass of CPD lesions after exposure of cells to UV radiation. Interestingly, we found that MSH2 can additionally regulate Polκ and REV1 focus formation in a PCNA-mUb-independent manner. These results reveal a novel role of MSH2 in post-UV cellular responses.

## MATERIALS AND METHODS

### Plasmids and reagents

Full-length mPolκ and mREV1 cDNAs were cloned into pEGFP-C3 (Clontech) or p3×Flag-CMV-14 (Sigma) expression vectors to generate eGFP or Flag fusion proteins. Flag-MSH2 plasmid was a kind gift from Dr Haiying Hang, Institute of Biophysics, Chinese Academy of Sciences.

Anti-Flag M2 agarose affinity gel and mouse monoclonal antibody against β-actin or Flag were purchased from Sigma (St Louis, MO, USA). Polyclonal antibodies against MSH2 and MSH6 were from the Bethyl Laboratories (Montgomery, TX, USA). Antibodies against RPA32 and Rad18 were from Abcam. Antibody against γH2AX was from the Cell Signaling Technology (Danvers, MA, USA). Antibody against CPD was from Cosmo Bio Co (Tokyo, Japan). Monoclonal antibodies against PCNA (PC10) and MSH2 were from Santa Cruz Biotechnology. Antibody against GFP was from Covance. Alexa Fluo-conjugated secondary antibodies were from Invitrogen.

### Cell Culture

Human HCT116, U2OS and 293T cells were obtained from the American Type Culture Collection (Rockville, MD, USA). LoVo cell was purchased from the Cell Resource Centre, Institute of Basic Medical Sciences, Chinese Academy of Medical Sciences. Rad18 stable knockdown U2OS cells were prepared as described (33). All cell lines were maintained in Dulbecco Modified Eagle medium supplemented with glutamax (Invitrogen) and 10% fetal bovine serum, 100 U/ml penicillin and 100 μg/ml streptomycin at 37°C in the presence of 5% CO_2_ if not specified. For transient transfection experiments, cells were transfected with accordingly constructs as indicated, using Fugene 6 (Roche) or Lipofectamin 2000 (Invitrogen) following the manufacturer’s protocol. Forty-eight hours later, transfected cells were harvested for further analysis.

### Mass spectrometry

HEK293T cells were transfected with a 3xFlag-Polκ construct and UV irradiated 40 h later. Cells were harvested 4 h following UV irradiation (15 J/m^2^) and cross-linked with 10 mM dimethyl dithiobispropionimidate. After treatment with 0.08% Triton X-100 in buffer [10 mM Tris–HCl (pH 8.0), 150 mM NaCl, 1 mM MgCl_2_, 10 mM ZnCl_2_] for 5 min, the Triton-insoluble fractions were harvested and incubated with anti-Flag M2 agarose to immunopurify 3xFlag-Polκ. The bound proteins were eluted with 250 μg/ml FLAG peptide (Sigma) in phosphate buffered saline (PBS). Eluted proteins were resolved by SDS–PAGE and revealed by silver staining. The apparent bands were excised, in-gel digested with trypsin and analyzed by liquid chromatography coupled with tandem mass spectrometry (LC-MS/MS) on a ProteomeX-LTQ mass spectrometer (Thermo Fisher Scientific, Waltham, USA) to identify the protein bands.

### Co-immunoprecipitation and western blotting

HEK293T cells transfected with peGFP-C3-mREV1 or peGFP-C3-mPolκ and pCMV-Flag-MSH2 were harvested, and the whole-cell lysates were immunoprecipitated with anti-Flag M2 agarose as described previously (34). U2OS cells were irradiated with UVC (15 J/m^2^), and chromatin-fractions were harvested as previously described (18). Samples were separated by SDS–PAGE and detected by immunoblotting with indicated antibodies.

### RNA interference

The introduction of small interfering RNA (siRNA) into U2OS cells was carried out with RNAiMAX (Invitrogen). Briefly, cells were exposed to 100 nM siRNA overnight in the presence of serum, followed by a change in medium the next morning. Whole-cell lysates and chromatin-fractions were harvested at 72 h after siRNA transfection. For foci formation assay, cells were further transfected with GFP-REV1 or GFP-Polκ at 48 h after siRNA transfection.

siRNAs directed against *MSH2* or *MSH6* were obtained from GenePharma (Shanghai, China). The gene-specific target sequences were as follows: *MSH2-1* (UAUAAGGCUUCUCCUGGCAAU), *MSH2-2* (UCCAGGCAUGCUUGUGUUGAA), *MSH2-3* (CCCAUGGGCUAUCAACUUAAU), *MSH2* (3′-UTR) (CCAGUAAUGGAAUGAAGGUAA) and *MSH6* (AGGCGAAGUAGCCGCCAAAUA). The negative control siNC sequence (UUCUCCGAACGUGUCACGU) was obtained from GenePharma. Unless otherwise specified, *MSH2-1* was used as the representative siRNA against *MSH2* in all experiments. Western blots were used to validate the knockdown ability of these siRNAs.

### Immunofluorescence

Cells were cultured on glass coverslips. Before fixing in 4% paraformaldehyde, the cells were washed in PBS once. For γH2AX, RPA2 and Rad18 staining, samples were treated with 0.5% Triton X-100 for 5–30 min followed by blocking with 5% fetal bovine serum and 1% goat serum for 1 h. After the block, cells were incubated with anti-γH2AX or anti-RPA2 or anti-Rad18 for 45 min. Then the samples were washed three times with PBS and incubated with the appropriate goat anti-mouse Alexa Fluor dye conjugated secondary antibody (Molecular Probes) for 45 min. The cells were then washed with PBS, counterstained with 4′,6-diamidino-2-phenylindole (DAPI) to visualize nuclear DNA. Images were acquired with a Leica DM5000 (Leica) equipped with HCX PL S-APO 63 × 1.3 oil CS immersion objective (Leica) and processed with Adobe Photoshop 7.0.

For quantitative analysis of UV-induced REV1 and Polκ foci formation, U2OS cells transfected with GFP-REV1 or GFP-Polκ were treated with UVC (15 J/m^2^) and fixed with 4% paraformaldehyde 8–12 h later after UV irradiation as described previously (15,18). Images were acquired using a Leica DM5000 (Leica) equipped with HCX PL S-APO 63 × 1.3 oil CS immersion objective (Leica). A minimum of 200 nuclei was analyzed for each treatment. For co-localization assay of UV-induced Polκ and MSH2 foci formation, U2OS cells transfected with GFP-Polκ were treated with UVC (15 J/m^2^) and fixed with 4% paraformaldehyde 8 h later. Cells were incubated with anti-MSH2 for 45 min. Then, the samples were washed with PBS and incubated with the appropriate goat anti-mouse Alexa Fluor dye conjugated secondary antibody (Molecular Probes) for 45 min. The cells were then washed with PBS, counterstained with DAPI to visualize nuclear DNA. Images were acquired with a Leica TCS SP5 equipped with HCX PL APO 63 × 1.4 oil CS immersion objective and processed with Adobe Photoshop 7.0.

### Immunofluorescent detection of unreplicated CPDs in S phase cells

Detection of CPDs in ssDNA templates was performed as previously described (35). Cells cultured on coverslips were irradiated with 0 or 5 J/m^2^ UVC light, followed by 30 min pulse-labeling with 10 μM 5-ethynyl-2-deoxyuridine (EdU) (Invitrogen) in the dark. Four hours later, cells were treated with 1% Triton-X100 in PBS for 2 min and subsequently fixed in 2% Formaldehyde/PBS containing 0.5% Triton-X100 for 15 min at room temperature. EdU-positive cells were visualized using AlexaFluor 647-conjugated azide following the manufacturer’s recommendations (Click-iTTM Edu imaging kit, Invitrogen). Unreplicated photoproducts were identified in non-denatured DNA using primary mouse monoclonal antibodies against CPDs (TDM2, CosmoBio). After incubation with secondary Alexafluor 488-labeled goat-anti-mouse antibodies (Molecular Probes, Inc.), nuclei were stained with DAPI. To check the functionality of the primary antibodies, immunofluorescence was performed essentially as described earlier in the text, except that after EdU detection, cells were fixed with 2% formaldehyde and denatured with 2 N HCl for 10 min. Images were analyzed by fluorescent microscopy.

## RESULTS

### MSH2 protein physically associates with Polκ and REV1

**Figure 1.**
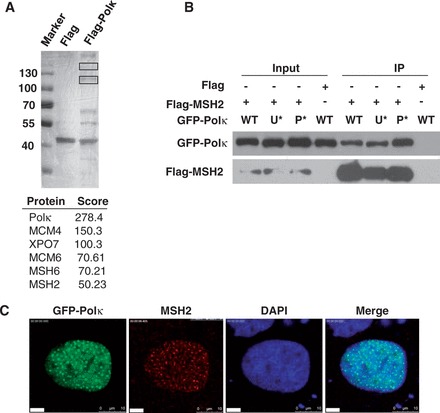
MSH2 physically interacts with Polκ. (**A**) Identification of MSH2 as a Polκ-associated protein. HEK 293T cells were transfected with a 3× Flag-Polκ expression vector and UV irradiated (15 J/m^2^). The cells were cross-linked, and lysates were harvested at 4 h after UV irradiation and immunoprecipitated with an anti-Flag M2 agarose affinity gel as described in ‘Materials and Methods’ section. The affinity-purified proteins associated with FLAG-Polκ were separated by SDS–PAGE and revealed by silver staining. The bands (indicated with black rectangles) were excised and analyzed via mass spectrometric sequencing. The results from mass spectrometry analysis were shown below. (**B**) Co-IP experiment showing the interaction between MSH2 and Polκ. Flag or Flag-MSH2 and GFP-Polκ or its mutants were co-transfected into 293T cells, and the lysates were immunoprecipitated using an anti-Flag M2 agarose affinity gel. The immunoprecipitate was then examined via western blot using anti-GFP antibody (Top panel) or anti-Flag antibody (Bottom panel). The input included 2% of the cell lysate used. WT represents wild-type GFP-Polκ, U* represents GFP-Polκ-UBZ*, P* represents GFP-Polκ-PIP*. (**C**) Co-localization of MSH2 and GFP-Polκ after UV irradiation. GFP-Polκ-transfected U2OS cells were UVC irradiated (15 J/m^2^) and further incubated for 8 h. The cells were then fixed and immunostained with anti-MSH2 monoclonal antibody and DAPI-stained.

To identify novel proteins that may regulate Polκ function *in vivo*, we transfected HEK293T cells with a 3 × Flag-Polκ expression vector and performed immunopurification using the triton-insoluble fraction of UV-irradiated cells. Affinity-purified proteins associated with FLAG-Polκ were separated by SDS–PAGE and revealed by silver staining (Figure 1A). Two indicated bands not observed in the affinity-purified control extracts were isolated from the gel, digested and analyzed with LC-MS/MS. This analysis revealed that several proteins copurified with Polκ, including MSH2, a protein essential for MMR. To confirm this interaction, Flag-MSH2 and GFP-Polκ were transiently transfected into 293 T cells and co-immunoprecipitation assays were performed. GFP-Polκ bound to Flag-MSH2 but not Flag protein alone (Figure 1B). Additionally, we found that mutation of either ubiquitin binding zinc finger motifs (UBZs) or PCNA binding peptide (PIP) in Polκ did not abolish the interaction, suggesting that the UBZs and PIP domains are not essential for the interaction. Consistently, we observed that Polκ and MSH2 co-localized at nuclear foci after exposure of cells to UV radiation (Figure 1C).

We also detected the interaction between REV1 and MSH2 by co-immunoprecipitation. GFP-REV1 and Flag-MSH2 were expressed in 293T cells, and cell lysates were incubated with anti-Flag M2 agarose. GFP-REV1 protein was co-immunoprecipitated from the extracts expressing both GFP-REV1 and Flag-MSH2, but not from the extracts expressing GFP-REV1 and Flag (Supplementary Figure S1A). Interestingly, GFP-REV1-UBM* (a REV1 mutant in which the ubiquitin-binding motifs [UBMs] are mutated) also interacted with MSH2, indicating that the UBMs in REV1 are not required for its association with MSH2. The interaction between REV1 and MSH2 was also confirmed by immunoprecipitation of Flag-REV1 in Flag-REV1-expressed cells and detection of endogenous MSH2 (Supplementary Figure S1B).

### MSH2 depletion impairs the association of Polκ and REV1 with stalled replication factories in cells exposed to UV radiation

**Figure 2.**
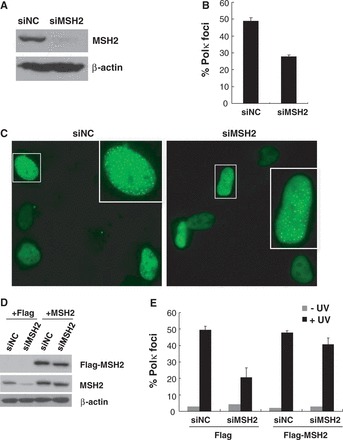
MSH2 is required for optimal Polκ focus formation after UV irradiation. (**A**) U2OS cells were transfected with siMSH2 or siNC, and the cell lysates were harvested 72 h later and separated by SDS–PAGE. The levels of MSH2 were detected by immunoblotting with anti-MSH2 antibodies. The lower panel is a loading control in which the blot was stripped and reprobed with a ß-actin antibody**.** (**B** and **C**) Polκ focus formation after UV irradiation. MSH2-depleted U2OS cells were transfected with GFP-Polκ. Cells were irradiated with 10 J/m^2^ UVC radiation and further incubated for 8 h. The cells were fixed and the proportion of GFP-Polκ expressing cells with greater than 30 foci was determined (B). All experiments were carried out in triplicate. Error bars indicate the standard error. (C) Representative fluorescence images of Polκ focus formation in cells transfected with siMSH2 or siNC. (**D** and **E**) U2OS cells were transfected with siMSH2 (3′-UTR) or siNC. Forty-eight hours later, the cells were further complemented with either Flag or Flag-MSH2. (D) The cell lysates were harvested 40 h later and separated by SDS–PAGE. The levels of MSH2 were detected by immunoblotting with anti-Flag or anti-MSH2 antibodies. The bottom panel is a loading control in which the blot was stripped and reprobed with a ß-actin antibody**.** (E) Polκ focus formation in U2OS/siMSH2 (3′-UTR)/Flag-MSH2 cells after UV irradiation were measured as described in (B).

To understand the significance of the MSH2/Polκ interaction *in vivo*, we asked whether depletion of MSH2 affects Polκ accumulation after UV irradiation. We transfected GFP-Polκ into MSH2-depleted U2OS cells and exposed the cells to 15 J/m^2^ UVC radiation (Figure 2A). Twelve hours later, the cells were fixed and analyzed for Polκ focus formation (Figure 2B and C). The proportion of GFP-Polκ expressing cells with greater than 30 foci (which were counted as foci positive cells) was significantly reduced when MSH2 was depleted (27.1%) compared with cells transfected with non-targeting siRNA (siNC) (49.5%). Similarly, the percentage of REV1 expressing cells with >30 foci decreased significantly after depletion of MSH2 (22.3%) compared with control cells transfected with siNC (42.7%) (Supplementary Figure S2A). UV radiation-induced REV1 focus formation was also impaired in cells treated with two other MSH2 siRNAs (Supplementary Figure S2B). We therefore conclude that the effect of MSH2 on the recruitment of Polκ and REV1 to sites of UV radiation-induced stalled replication forks is not the result of siRNA off-target effects.

To further confirm that reduced Polκ focus formation observed in MSH2-depleted cells following UVC exposure results from loss of MSH2, pCMV-Flag-MSH2 and pEGFP-Polκ constructs were co-transfected into cells depleted of MSH2 by siMSH2 (3′-UTR), which targets the 3 prime untranslated region (3′-UTR) of this mismastch repair protein (Figure 2D). Expression of Flag-MSH2 significantly increased UV radiation-induced Polκ focus formation in MSH2-depleted cells (Figure 2E). The defect in UV radiation-induced REV1 focus formation was largely restored in MSH2-depleted cells reconstituted with MSH2, but not with Flag (Supplementary Figure S2C), suggesting that MSH2 regulates Polκ and REV1 recruitment after exposure of cells to UV radiation. Furthermore, we found that depletion of MSH6, a partner of MSH2 bound to UV adducts, also significantly reduced Polκ and REV1 foci formation (Supplementary Figure S3).

### MSH2 regulates UV-induced PCNA monoubiquitination

**Figure 3.**
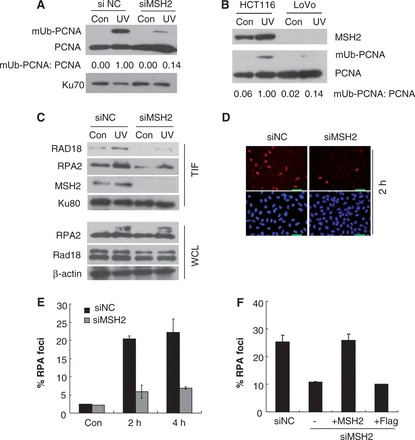
Depletion of MSH2 led to a reduced level of PCNA-mUb and RPA foci formation after UV exposure. (**A**) U2OS cells were transfected with siMSH2 or siNC and incubated for 72 h. Then, the cells were irradiated with 15 J/m^2^ UVC and further incubated for 4 h. The triton-insoluble fractions were harvested and separated by SDS–PAGE. Levels of unmodified PCNA (PCNA) and mUb-PCNA were analyzed by western blot with anti-PCNA antibody and quantified using ImageJ. The levels of Ku 70 were analyzed as loading controls. (**B**) HCT116 and LoVo cells were UV irradiated and the triton-insoluble fractions were harvested and analyzed as in (A). (**C**) U2OS cells were transfected with siMSH2 or siNC and incubated for 72 h. Then, the cells were irradiated with 15 J/m^2^ UVC and further incubated for 2 h. The triton-insoluble fractions (TIF) were harvested and separated by SDS–PAGE. The levels of Rad18 and RPA2 were detected by immunoblotting with anti-Rad18 or anti-RPA2 antibodies. The levels of Ku 80 were analyzed as loading controls. The levels of Rad18 and RPA2 in whole-cell lysates (WCL) were also detected. The levels of ß-actin were analyzed as WCL loading controls**.** (**D**) Representative images of cells stained with DAPI or antibody against RPA2 after UV irradiation. U2OS cells were transfected with siMSH2 or siNC and incubated for 72 h. Then, the cells were irradiated with 5 J/m^2^ UVC and cultured further. At the indicated time points, cells were pre-extracted with 0.5% Triton X-100 for 30 min and then fixed, immunostained with anti-RPA2 antibody and DAPI-stained. (**E**) Quantification of the percentage of cells with RPA2 foci. For each cell at each time point, at least 250 cells were counted, and the percentage of cells with over 10 RPA2 foci was determined. The results are the average of two independent experiments. Error bars represent standard error. (**F**) U2OS/siMSH2 (3′-UTR) cells were transfected with Flag-MSH2 or Flag constructs and RPA2 foci formation after UV irradiation were measured as described in (D) and (E).

As the recruitment of Polκ to stalled replication forks after exposure of cells to UV radiation is mediated through mUb-PCNA, we asked whether depletion of MSH2 affects the generation of UV radiation-induced PCNA-mUb. We transfected U2OS cells with siMSH2 and exposed the MSH2-depleted cells to UV light. The chromatin fractions of these cells were collected and separated on SDS–PAGE gels. An obvious reduction of PCNA-mUb was detected in MSH2-depleted cells relative to that in siNC-transfected cells (Figure 3A). To determine whether this phenomenon resulted from defective MMR in MSH2-depleted cells, we compared the extent of UV-induced PCNA-mUb formation in MMR-deficient LoVo cells (MSH2-deficient) and HCT116 cells (MLH1-deficient). An obvious decrease was still observed (Figure 3B), suggesting that at least partial reduction of UV-induced PCNA-mUb formation triggered by MSH2 depletion was not related to MMR. To confirm this conclusion, we examined the effect of MSH2 depletion on post-UV PCNA-mUb in HCT116 cells. An obvious reduction in the level of PCNA-mUb was observed in MSH2-depleted HCT116 cells relative to controls at 4 h after exposure to UV irradiation (Supplementary Figure S4).

### MSH2 regulates UV-induced Rad18 and RPA foci formation

To further explore the reduction of PCNA-mUb levels under conditions of MSH2 depletion, we examined the amount of chromatin-bound Rad18 (the E3 ligase for PCNA-mUb) following exposure of cells to UV radiation. An unambiguous reduction of chromatin-bound Rad18 was detected by 2 h after UV irradiation (Figure 3C). UV-induced Rad18 focus formation was also substantially impaired by MSH2 depletion (Supplementary Figure S5). Rad18 is recruited to blocked replication forks by virtue of its affinity for RPA-coated ssDNA (22). We therefore measured the level of chromatin-bound RPA in MSH2-depleted cells and observed a marked reduction compared with that in siNC-transfected cells (Figure 3C). In contrast, the total cellular abundance of RPA was unchanged. We also examined focus formation by RPA in UV-irradiated MSH2-depleted cells. At 2 h and 4 h after irradiation, the cells were treated to remove unbound RPA and were then fixed and stained to reveal chromatin-bound RPA by immunofluorescence (32). As seen in Figure 3E, RPA foci in the nuclei of unirradiated U2OS cells were scarce. At 2 h after UV irradiation (5 J/m^2^), ∼20.4% of nuclei examined exhibited robust formation of RPA foci in siNC-transfected cells (Figures 3D and E). In contrast, only ∼5.9% of cells transfected with siMSH2 displayed UV-induced RPA foci. A similar difference was detected in these cells at 4 h after UV exposure (Figure 3E). To confirm that the defect in RPA focus formation after UV irradiation is a specific consequence of MSH2 depletion, we attempted to rescue this defect by esxpressing Flag-MSH2 in cells depleted of MSH2 by transfection with siMSH2 (3′-UTR). The siMSH2 (3′-UTR) also elicited a defect in UV-induced RPA focus formation (10.6%) versus the control (23.0%) at 2 h after UV irradiation (5 J/m^2^) (Figure 3F). Notably, reconstitution with Flag-MSH2 but not Flag alone increased UV radiation-induced RPA focus formation in these MSH2-depleted cells (Figure 3F). Hence, the reduction in PCNA-mUb levels in MSH2-depleted cells after UV exposure is likely caused by impaired RPA chromatin loading.

### MSH2 also modulates UV-induced Polκ focus formation in a PCNA monoubiquitination-independent fashion

**Figure 4.**
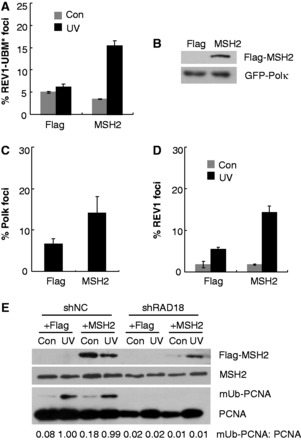
MSH2 modulates UV-induced Polκ and REV1 foci formation in a PCNA monoubiquitination-independent fashion. (**A**) U2OS cells were co-transfected with GFP-REV1-UBM* and Flag-MSH2 or Flag constructs and then UV irradiated with 10 J/m^2^ UVC. Eight hours later, cells were fixed and the proportion of GFP-REV1-UBM* expressing cells in which the protein was localized in nuclear foci was determined. (**B** and **C**) Rad18 stable knockdown cells were co-transfected with GFP-Polκ and Flag-MSH2 or Flag constructs. (B) Western blots showing expression of Flag-MSH2 (top panel) and GFP-Polκ (bottom panel) in Rad18 stable knockdown cell line. (C) The cells were UV irradiated with 10 J/m^2^ UVC. Eight hours later, cells were fixed and the proportion of GFP-Polκ expressing cells with >30 foci was determined. Error bars indicate the standard error from three different experiments. (**D**) Rad18 stable knockdown cells were co-transfected with GFP-REV1 and Flag-MSH2 or Flag constructs and then UV irradiated with 10 J/m^2^ UVC. Eight hours later, cells were fixed and the proportion of GFP-REV1 expressing cells in which the protein was localized in nuclear foci was determined. The experiments were carried out in triplicate. Error bars indicate the standard error. (**E**) Control and Rad18 stable knockdown cells were transfected with Flag-MSH2 or Flag constructs. Eight hours after UVC irradiation (15 J/m^2^), the triton-insoluble fractions were harvested and analyzed as in Figure 3A.

As mutation of REV1 UBMs abolishes its enhanced interaction with mUb-PCNA (17) but does not affect its association with MSH2, we examined the possibility that MSH2 regulates REV1 focus formation in a PCNA-mUb-independent manner. We expressed MSH2 and GFP-REV1-UBM* in U2OS cells and monitored the focus formation of REV1 mutant after UV irradiation (Figure 4A). Expression of MSH2-Flag but not Flag alone increased UV-induced REV1-UBM* focus formation. As mutation of the UBMs in REV1 abolishes its enhanced interaction with mUb-PCNA (17), the increased formation of REV1-UBM* foci imparted by MSH2 expression suggests that MSH2 may regulate UV-induced REV1 focus formation in a PCNA-mUb-independent manner. Notably, expression of MSH2 does not induce post-UV Polκ-UBZ* focus formation, possibly a consequence of the essential role of the UBZ motifs in this process (15).

To further confirm that MSH2 can additionally mediate UV-induced Polκ and REV1 focus formation in a PCNA-mUb-independent fashion, we co-transfected Flag-MSH2 and GFP-Polκ or GFP-REV1 constructs into a previously established Rad18 stable knockdown cell line (33) and then exposed the cells to UV light. As shown in Figures 4B and C, Polκ focus formation was enhanced in cells expressing Flag-MSH2 compared with controls expressing Flag alone. Similarly, REV1 focus formation was increased in Rad18-depleted cells expressing Flag-MSH2 compared with cells expressing Flag (Figure 4D). To exclude the possibility that increased Polκ and REV1 foci formation results from enhanced PCNA-mUb levels after expression of MSH2, Rad18-depleted cells expressing Flag-MSH2 were exposed to UV irradiation (15 J/m^2^), and the chromatin fractions were harvested. As expected, depletion of Rad18 led to a significant reduction of PCNA-mUb in the UV-irradiated cells (Figure 4E). Importantly, expression of exogenous MSH2 protein did not increase the level of PCNA-mUb in cells with or without Rad18 depletion (Figure 4E). Considering that the expression level of exogenous MSH2 in Rad18-depleted cells is modest, we additionally transfected the Flag-MSH2 construct into 293T cells and noted that over-expression of exogenous MSH2 did not lead to detectable enhancement of PCNA-mUb after exposure to UV radiation (Supplementary Figure S6).

Hence, it is distinctly possible that impaired focus formation of Polκ and REV1 in MSH2-depleted cells after exposure to UV radiation results from defective PCNA-mUb function as well as an attenuated interaction between MSH2 and Polκ or REV1.

### MSH2 regulates UV-induced CPD lesion bypass

**Figure 5.**
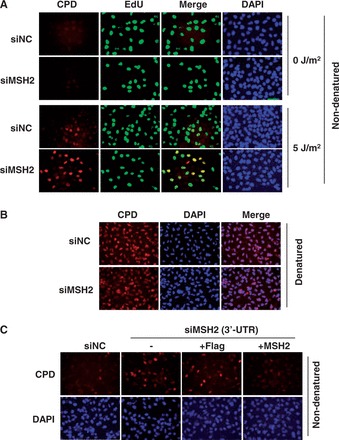
MSH2 is required for gap filling opposite genomic CPD lesions. (**A**) MSH2-depleted U2OS cells were exposed to 5 J/m^2^ UVC and immediately pulse-labeled with EdU. Four hours later, cells were fixed and immunostained for CPDs (red) in ssDNA of nuclei (DAPI, blue) in replicating (EdU-positive, green) and non-replicating cells at the time of UVC exposure. Merge represents combined stainings for CPD lesions in ssDNA and for EdU. ‘Non-denaturated’ means no denaturation of DNA with HCl treatment during CPD immunostaining. (**B**) MSH2-depleted U2OS cells were exposed to 5 J/m^2^ UVC. Four hours later, cells were fixed and immunostained for CPDs (red) in nuclei (DAPI, blue). Merge represents combined stainings for CPD lesions in DNA and for nuclei. ‘Denaturated’ means denaturation of DNA with HCl treatment during CPD immunostaining. (**C**) U2OS cells transfected with siMSH2 (3′-UTR) were further complemented with either Flag or Falg-MSH2 and then exposed to 5 J/m^2^ UVC. Four hours later, cells were fixed and immunostained for CPDs (red) in ssDNA of nuclei (DAPI, blue).

It has been reported that unreplicated CPDs persist longer in PCNAK164R mouse embryonic fibroblasts (defective in TLS) than in control cells. To provide further evidence of a contribution of MSH2 to UV radiation-induced TLS in normal cells, we examined the ability of MSH2-depleted U2OS cells to bypass UV-induced CPD lesions in replicating cells. The cells were exposed to 5 J/m^2^ UVC light and pulse-labeled with EdU, thereby facilitating the identification of cells that were replicating during UVC exposure. Using an experimental approach recently developed to monitor the disappearance of CPDs in ssDNA (35), we observed scanty CPD positive signals in the nuclei of unirradiated cells and in replicating siNC-transfected cells 4 h after UVC treatment, suggesting efficient bypass across these lesions (Figure 5A). In contrast, in nuclei of EdU-positive cells deficient for MSH2, unreplicated CPDs were clearly detectable in non-denatured DNA at 4 h after UVC exposure. Given that MSH2 is not directly involved in the excision of CPDs (36,37) and that equivalent amounts of CPDs were detected in denatured DNA in cells transfected with either siMSH2 or siNC (Figure 5B), the strong signal of CPDs in ssDNA in MSH2 knockdown cells strongly suggests that MSH2 is required for gap filling opposite genomic CPD lesions. Similarly, a strong signal for CPDs in ssDNA was also detected in U2OS cells transfected with a siMSH2 (3′-UTR) (Figure 5C). To further confirm the defect was imparted by MSH2 depletion, we performed a rescue experiment and determined that complementation with Flag-MSH2 (but not Flag) can reverse the intense signal of CPDs in ssDNA in MSH2-depleted cells (Figure 5C).

## DISCUSSION

Persistent arrested DNA replication threatens the viability of dividing cells. TLS can use specialized DNA polymerases to catalyze DNA synthesis past sites of base damage, thereby alleviating the threat of cell death, but at the expense of an increased mutation frequency. In view of the crucial role that functional specialized DNA polymerases play in spontaneous and induced mutagenesis *in vivo* (3), a detailed understanding of the events that regulate their recruitment to stalled replication forks is fundamental.

In the present study, we determined that Polκ and REV1 interact with MSH2, a protein that is required for normal MMR. MMR proteins are integral to the maintenance of genomic stability and suppression of tumorigenesis due to their role in the repair of post-replicative errors. MSH2 can heterodimerize with MSH6 or MSH3 and can play a critical role in the recognition and signaling of MMR (38). However, the MSH2-MSH6 complex has also been reported to be an S phase component of replication centers independent of mispaired bases (39). Some nuclear foci formed by MSH2-MSH6 do not necessarily represent sites of active MMR (39), suggesting that MSH2 has a role beyond canonical MMR *in vivo*. More recently, Pena-Diaz *et al.* reported the existence of a non-canonical MMR pathway in a variety of cell types. This process, in which the MMR pathway is activated by various DNA lesions rather than by mispairs, is largely independent of DNA replication, lacks strand directionality, triggers PCNA-mUb and promotes recruitment of the error-prone polymerase-η to chromatin (40).

Given that the MSH2-MSH6 heterodimer has been shown to bind to UV radiation-induced adducts (41), we asked whether depletion of MSH2 affects the recruitment of Polκ to damaged sites after UV irradiation. Our data indicate that depletion of MSH2 impairs the formation of Polκ and REV1 foci after exposure of cells to UV radiation. Further analysis revealed that knockdown of MSH2 decreased PCNA-mUb formation after UV exposure, comparable with its reported role in the repair of oxidative base damage (33,42). This result is not consistent with a recent report that states that the impaired effect of MSH2 depletion on PCNA-mUb is restricted to conditions of oxidative stress (42). In that report, non-replicating primary fibroblasts were used (42), whereas our experiments used replicating cells with active TLS. Conceivably, differences in cell cycle stages may explain the apparent discrepancy. Intriguingly, we found that modulation of MSH2 on PCNA-mUb also held true after treatment of cells with the alkylating agent methylmethanesulfonate (data not shown), suggesting that MSH2 may regulate PCNA ubiquitination under diversified situations.

In our hands, the impaired effect of MSH2 depletion on UV-induced PCNA-mUb was detectable in MMR-deficient HCT116 cells. Given that the endonuclease activity of MLH1/PMS2 is required for ncMMR-triggered PCNA-mUb (40), our data suggest that at least a partial reduction in PCNA-mUb levels is unrelated to either canonical or non-canonical MMR. This is in line with the fact that deficiency in MSH2 but not in MLH1 has an effect on somatic hypermutation. Consistently, expression of a MMR-deficient MSH2 missense mutation (MSH2-A834T) found in hereditary non-polyposis colorectal cancer families (43) is able to rescue REV1 focus formation in MSH2-depleted cells (Supplementary Figure S7), suggesting that the role of MSH2 in MMR and TLS could be separable, which might be useful in the development of novel strategies to reduce unwanted mutagenesis that promotes tumor resistance.

We also observed that MSH2 is required for optimal UV-induced chromatin loading and focus formation of Rad18 and RPA. Combined with the fact that MSH2 can bind to UV lesions, it is plausible that this protein plays a crucial role in the detection of UV radiation-induced lesions and initiates the early signaling events leading to the formation of PCNA-mUb. Similarly, MSH2 but not MLH1 has been reported to be involved in MMC-induced FancD2-mUb (44,45). Hence, the reduced post-UV formation of Polκ and REV1 foci imparted by MSH2 depletion is possibly attributable to impaired PCNA-mUb activity. In support of this notion, PCNA-mUb is required for the majority of A->T mutations generated downstream of MSH2 during somatic hypermutation (46,47).

Compelling data indicate that TLS polymerases can be recruited to damaged DNA in the absence of PCNA-mUb (32). In agreement with this notion, we found that expressing MSH2 can increase UV radiation-induced focus formation in a REV1 mutant in which the UBMs are mutated, and consequently their enhanced association with mUb-PCNA is significantly abolished (17). Thus, MSH2 might also regulate the recruitment of specialized DNA polymerases to sites of UV radiation-induced DNA damage in a PCNA-mUb-independent fashion. Consistent with this suggestion, expression of MSH2 in stable Rad18 knockdown cells enhanced Polκ and REV1 focus formation without a visible increase of PCNA-mUb.

The specific mechanism of PCNA-mUb-independent polymerase recruitment mediated by MSH2 remains to be established. The interaction between MSH2 and Polκ or REV1 might play a direct role in this process. However, we cannot exclude other possibilities. For example, MSH2 might regulate post-UV chromatin loading of the Fanconi anemia core complex, further modulating REV1 focus formation (48).

Our results hint at the possibility that MSH2 may act as a sensor of UV radiation-induced DNA damage *in vivo* thereby modulating the recruitment of specialized polymerases through PCNA-mUb-dependent and -independent mechanisms.

MSH2 has been previously implicated in post-UV cellular responses (37). MSH2-null mice are more susceptible to UVB-induced skin cancer due to a loss of UVB-induced apoptosis (37,49). Despite the fact that MSH2 protein can bind to UV photoproducts, this protein has not been implicated in the excision repair of UV radiation-induced DNA damage (36,37), which is also confirmed by the result that equivalent amounts of CPDs were detected in denatured DNA in cells transfected with either siMSH2 or siNC. Our observation that a strong CPD signal in ssDNA is detected in MSH2-depleted cells indicates that MSH2 is required for optimal TLS. Combined with the fact that PCNA-mUb is critical for a TLS pathway that replicates CPD photoproducts (35) and that MSH2 is required for optimal PCNA-mUb formation after UV irradiation, the involvement of MSH2 in binding to UV radiation-induced DNA lesions and modulating PCNA-mUb might prove to be an essential role for MSH2 in TLS. Further studies will be required to reveal the contribution of MSH2 in UV radiation-induced TLS and associated mutagenesis. Given that MSH2 is also apparently required for the rapid generation of PCNA-mUb after exposure of cells to hydrogen peroxide, as well as FANCD2 monoubiquitination and focus formation following treatment of cells with mitomycin C (33,42,44), it is conceivable that MSH2 plays multiple roles in the maintenance of genome stability.

## SUPPLEMENTARY DATA

Supplementary Data are available at NAR Online.

## FUNDING

National Basic Research Program of China [2011CB944302, 2013CB945000, 2012CB944702]; Natural Science Foundation of China [30970588, 31170730, 81371415, 31300880]; Chinese Academy of Sciences ‘One-Hundred-Talent Program’; State Key Laboratory of Biomembrane and Membrane Biotechnology. Funding for open access charge: National Basic Research Program of China [2011CB944302, 2013CB945000].

*Conflict of interest statement*. None declared.

## Supplementary Material

Supplementary Data

## References

[1] Friedberg EC (2005). Suffering in silence: the tolerance of DNA damage. Nat. Rev. Mol. Cell Biol..

[2] Yang W, Woodgate R (2007). What a difference a decade makes: insights into translesion DNA synthesis. Proc. Natl Acad. Sci. USA.

[3] Guo C, Kosarek-Stancel JN, Tang TS, Friedberg EC (2009). Y-family DNA polymerases in mammalian cells. Cell Mol. Life. Sci..

[4] Lehmann AR, Niimi A, Ogi T, Brown S, Sabbioneda S, Wing JF, Kannouche PL, Green CM (2007). Translesion synthesis: Y-family polymerases and the polymerase switch. DNA Repair (Amst.).

[5] Zhang Y, Yuan F, Wu X, Wang M, Rechkoblit O, Taylor JS, Geacintov NE, Wang Z (2000). Error-free and error-prone lesion bypass by human DNA polymerase kappa *in vitro*. Nucleic Acids Res..

[6] Masutani C, Kusumoto R, Yamada A, Dohmae N, Yokoi M, Yuasa M, Araki M, Iwai S, Takio K, Hanaoka F (1999). The XPV (xeroderma pigmentosum variant) gene encodes human DNA polymerase eta. Nature.

[7] Busuttil RA, Lin Q, Stambrook PJ, Kucherlapati R, Vijg J (2008). Mutation frequencies and spectra in DNA polymerase eta-deficient mice. Cancer Res..

[8] Ogi T, Shinkai Y, Tanaka K, Ohmori H (2002). Polkappa protects mammalian cells against the lethal and mutagenic effects of benzo[a]pyrene. Proc. Natl Acad. Sci. USA.

[9] Stancel JN, McDaniel LD, Velasco S, Richardson J, Guo C, Friedberg EC (2009). Polk mutant mice have a spontaneous mutator phenotype. DNA Repair (Amst.).

[10] Bi X, Slater DM, Ohmori H, Vaziri C (2005). DNA polymerase kappa is specifically required for recovery from the benzo[a]pyrene-dihydrodiol epoxide (BPDE)-induced S-phase checkpoint. J. Biol. Chem..

[11] Ogi T, Kato T, Kato T, Ohmori H (1999). Mutation enhancement by DINB1, a mammalian homologue of the Escherichia coli mutagenesis protein dinB. Genes Cells.

[12] Bavoux C, Leopoldino AM, Bergoglio V, O-Wang J, Ogi T, Bieth A, Judde JG, Pena SD, Poupon MF, Helleday T (2005). Up-regulation of the error-prone DNA polymerase {kappa} promotes pleiotropic genetic alterations and tumorigenesis. Cancer Res..

[13] Jones MJ, Colnaghi L, Huang TT (2011). Dysregulation of DNA polymerase kappa recruitment to replication forks results in genomic instability. EMBO J..

[14] Bienko M, Green CM, Crosetto N, Rudolf F, Zapart G, Coull B, Kannouche P, Wider G, Peter M, Lehmann AR (2005). Ubiquitin-binding domains in Y-family polymerases regulate translesion synthesis. Science.

[15] Guo C, Tang TS, Bienko M, Dikic I, Friedberg EC (2008). Requirements for the interaction of mouse Polkappa with ubiquitin and its biological significance. J. Biol. Chem..

[16] Plosky BS, Vidal AE, Fernandez de Henestrosa AR, McLenigan MP, McDonald JP, Mead S, Woodgate R (2006). Controlling the subcellular localization of DNA polymerases iota and eta via interactions with ubiquitin. EMBO J..

[17] Guo C, Tang TS, Bienko M, Parker JL, Bielen AB, Sonoda E, Takeda S, Ulrich HD, Dikic I, Friedberg EC (2006). Ubiquitin-binding motifs in REV1 protein are required for its role in the tolerance of DNA damage. Mol. Cell. Biol..

[18] Guo C, Sonoda E, Tang TS, Parker JL, Bielen AB, Takeda S, Ulrich HD, Friedberg EC (2006). REV1 protein interacts with PCNA: significance of the REV1 BRCT domain *in vitro* and *in vivo*. Mol. Cell.

[19] Bi X, Barkley LR, Slater DM, Tateishi S, Yamaizumi M, Ohmori H, Vaziri C (2006). Rad18 regulates DNA polymerase kappa and is required for recovery from S-phase checkpoint-mediated arrest. Mol. Cell. Biol..

[20] Hoege C, Pfander B, Moldovan GL, Pyrowolakis G, Jentsch S (2002). RAD6-dependent DNA repair is linked to modification of PCNA by ubiquitin and SUMO. Nature.

[21] Stelter P, Ulrich HD (2003). Control of spontaneous and damage-induced mutagenesis by SUMO and ubiquitin conjugation. Nature.

[22] Davies AA, Huttner D, Daigaku Y, Chen S, Ulrich HD (2008). Activation of ubiquitin-dependent DNA damage bypass is mediated by replication protein A. Mol. Cell.

[23] Huang TT, Nijman SM, Mirchandani KD, Galardy PJ, Cohn MA, Haas W, Gygi SP, Ploegh HL, Bernards R, D'Andrea AD (2006). Regulation of monoubiquitinated PCNA by DUB autocleavage. Nat. Cell. Biol..

[24] Soria G, Speroni J, Podhajcer OL, Prives C, Gottifredi V (2008). p21 differentially regulates DNA replication and DNA-repair-associated processes after UV irradiation. J. Cell Sci..

[25] Yanagihara H, Kobayashi J, Tateishi S, Kato A, Matsuura S, Tauchi H, Yamada K, Takezawa J, Sugasawa K, Masutani C (2011). NBS1 recruits RAD18 via a RAD6-like domain and regulates Pol eta-dependent translesion DNA synthesis. Mol. Cell.

[26] Centore RC, Yazinski SA, Tse A, Zou L (2012). Spartan/C1orf124, a reader of PCNA ubiquitylation and a regulator of UV-induced DNA damage response. Mol. Cell.

[27] Davis EJ, Lachaud C, Appleton P, Macartney TJ, Nathke I, Rouse J (2012). DVC1 (C1orf124) recruits the p97 protein segregase to sites of DNA damage. Nat. Struct. Mol. Biol..

[28] Juhasz S, Balogh D, Hajdu I, Burkovics P, Villamil MA, Zhuang Z, Haracska L (2012). Characterization of human Spartan/C1orf124, an ubiquitin-PCNA interacting regulator of DNA damage tolerance. Nucleic Acids Res..

[29] Kim MS, Machida Y, Vashisht AA, Wohlschlegel JA, Pang YP, Machida YJ (2013). Regulation of error-prone translesion synthesis by Spartan/C1orf124. Nucleic Acids Res..

[30] Ghosal G, Leung JW, Nair BC, Fong KW, Chen J (2012). Proliferating cell nuclear antigen (PCNA)-binding protein C1orf124 is a regulator of translesion synthesis. J. Biol. Chem..

[31] Krijger PH, van den Berk PC, Wit N, Langerak P, Jansen JG, Reynaud CA, de Wind N, Jacobs H (2011). PCNA ubiquitination-independent activation of polymerase eta during somatic hypermutation and DNA damage tolerance. DNA Repair (Amst.).

[32] Hendel A, Krijger PH, Diamant N, Goren Z, Langerak P, Kim J, Reissner T, Lee KY, Geacintov NE, Carell T (2011). PCNA ubiquitination is important, but not essential for translesion DNA synthesis in mammalian cells. PLoS Genet..

[33] Zhang X, Lv L, Chen Q, Yuan F, Zhang T, Yang Y, Zhang H, Wang Y, Jia Y, Qian L (2013). Mouse DNA polymerase kappa has a functional role in the repair of DNA strand breaks. DNA Repair (Amst.).

[34] Guo C, Fischhaber PL, Luk-Paszyc MJ, Masuda Y, Zhou J, Kamiya K, Kisker C, Friedberg EC (2003). Mouse Rev1 protein interacts with multiple DNA polymerases involved in translesion DNA synthesis. EMBO J..

[35] Temviriyanukul P, van Hees-Stuivenberg S, Delbos F, Jacobs H, de Wind N, Jansen JG (2012). Temporally distinct translesion synthesis pathways for ultraviolet light-induced photoproducts in the mammalian genome. DNA Repair (Amst.).

[36] Peters AC, Young LC, Maeda T, Tron VA, Andrew SE (2003). Mammalian DNA mismatch repair protects cells from UVB-induced DNA damage by facilitating apoptosis and p53 activation. DNA Repair (Amst.).

[37] Young LC, Hays JB, Tron VA, Andrew SE (2003). DNA mismatch repair proteins: potential guardians against genomic instability and tumorigenesis induced by ultraviolet photoproducts. J. Invest. Dermatol..

[38] Li GM (2008). Mechanisms and functions of DNA mismatch repair. Cell Res..

[39] Hombauer H, Campbell CS, Smith CE, Desai A, Kolodner RD (2011). Visualization of eukaryotic DNA mismatch repair reveals distinct recognition and repair intermediates. Cell.

[40] Pena-Diaz J, Bregenhorn S, Ghodgaonkar M, Follonier C, Artola-Boran M, Castor D, Lopes M, Sartori AA, Jiricny J (2012). Noncanonical mismatch repair as a source of genomic instability in human cells. Mol. Cell.

[41] Wang H, Lawrence CW, Li GM, Hays JB (1999). Specific binding of human MSH2.MSH6 mismatch-repair protein heterodimers to DNA incorporating thymine- or uracil-containing UV light photoproducts opposite mismatched bases. J. Biol. Chem..

[42] Zlatanou A, Despras E, Braz-Petta T, Boubakour-Azzouz I, Pouvelle C, Stewart GS, Nakajima S, Yasui A, Ishchenko AA, Kannouche PL (2011). The hMsh2-hMsh6 complex acts in concert with monoubiquitinated PCNA and Pol eta in response to oxidative DNA damage in human cells. Mol. Cell.

[43] Lutzen A, de Wind N, Georgijevic D, Nielsen FC, Rasmussen LJ (2008). Functional analysis of HNPCC-related missense mutations in MSH2. Mutat. Res..

[44] Williams SA, Wilson JB, Clark AP, Mitson-Salazar A, Tomashevski A, Ananth S, Glazer PM, Semmes OJ, Bale AE, Jones NJ (2011). Functional and physical interaction between the mismatch repair and FA-BRCA pathways. Hum. Mol. Genet..

[45] Huang M, Kennedy R, Ali AM, Moreau LA, Meetei AR, D'Andrea AD, Chen CC (2011). Human MutS and FANCM complexes function as redundant DNA damage sensors in the Fanconi anemia pathway. DNA Repair (Amst.).

[46] Langerak P, Nygren AO, Krijger PH, van den Berk PC, Jacobs H (2007). A/T mutagenesis in hypermutated immunoglobulin genes strongly depends on PCNAK164 modification. J. Exp. Med..

[47] Roa S, Avdievich E, Peled JU, Maccarthy T, Werling U, Kuang FL, Kan R, Zhao C, Bergman A, Cohen PE (2008). Ubiquitylated PCNA plays a role in somatic hypermutation and class-switch recombination and is required for meiotic progression. Proc. Natl Acad. Sci. USA.

[48] Mirchandani KD, McCaffrey RM, D'Andrea AD (2008). The Fanconi anemia core complex is required for efficient point mutagenesis and Rev1 foci assembly. DNA Repair (Amst.).

[49] Meira LB, Cheo DL, Reis AM, Claij N, Burns DK, te Riele H, Friedberg EC (2002). Mice defective in the mismatch repair gene Msh2 show increased predisposition to UVB radiation-induced skin cancer. DNA Repair (Amst.).

