# Supplementary material for: Mismatch repair protein MSH2 regulates translesion DNA synthesis following exposure of cells to UV radiation
Source: Nucleic Acids Res. 2013 Sep 12;41(22):10312–22. doi: 10.1093/nar/gkt793 (PMC3905884; doi:10.1093/nar/gkt793)
Supplement: Supplementary Data [file supp_41_22_10312__index.html]

Mismatch repair protein MSH2 regulates translesion DNA synthesis following exposure of cells to UV radiation — Mismatch repair protein MSH2 regulates translesion DNA synthesis following exposure of cells to UV radiation — Supplementary Data 

# Mismatch repair protein MSH2 regulates translesion DNA synthesis following exposure of cells to UV radiation

## Supplementary Data

files

**Files in this Data Supplement:**

- Supplementary Data - pdf file
