# Supplementary material for: Mismatch repair protein MSH2 regulates translesion DNA synthesis following exposure of cells to UV radiation
Source: Nucleic Acids Res. 2013 Sep 12;41(22):10312–22. doi: 10.1093/nar/gkt793 (PMC3905884; doi:10.1093/nar/gkt793)

SUPPLEMENTARY FIGURE LEGENDS

Fig. S1. MSH2 interacts with REV1. **(A)** Anti-Flag M2 agarose affinity gel was incubated with 293T cell lysates expressing GFP-REV1 wild-type (WT) or UBM* mutant (U*) and Flag-MSH2 or Flag (control) as indicated. Bound proteins were detected by western blot with anti-GFP or anti-Flag antibodies. **(B)** Anti-Flag M2 agarose affinity gel was incubated with 293T cell lysates expressing Flag-REV1 or Flag (control) as indicated. Bound proteins were detected by western blot with anti-MSH2 or anti-Flag antibodies. Inputs contain about 1/50 of the lysates used for the immunoprecipitations.

Fig. S2. MSH2 is required for optimal REV1 focus formation after UV irradiation. **(A)** REV1 focus formation after UV irradiation. MSH2-depleted U2OS cells were transfected with GFP-REV1 and then irradiated with 10 J/m^2 UVC radiation and further incubated for 8 hours. The proportion of GFP-REV1 expressing cells with more than 30 foci was determined (Left panel). Right panel: Representative fluorescence images of REV1 focus formation in cells transfected with siMSH2 or siNC. **(B)** U2OS cells were transfected with two other siMSH2 oligos or siNC. Left panel: The cell lysates were harvested at 50 h following transfection and separated by SDS-PAGE. The levels of MSH2 were detected by western blot with anti-MSH2 antibodies. Right panel: The cells were transfected with GFP-REV1 at 50 h following siRNA transfection. REV1 focus formation was measured as in **(A)**. **(C)** REV1 focus formation in U2OS/ shMSH2 (3’-UTR)/ Flag-MSH2 cells after UV irradiation. Left panel： U2OS cells were infected with either shMSH2 (3’-UTR) or shNC and selected with puromycin for about 7 days. The cell lysates were harvested and separated by SDS-PAGE. The levels of MSH2 were detected by western blot with anti-MSH2 antibodies. Right panel： U2OS/ shMSH2 (3’-UTR) cells were complemented with either Flag or Flag-MSH2 and REV1 focus formation were measured as in **(A)**.

Fig. S3. MSH6 is required for optimal TLS polymerase focus formation after UV exposure. **(A)** U2OS cells were transfected with siMSH6 or siNC and cell lysates were harvested 72 h later and separated by SDS-PAGE. The levels of MSH6 were detected by western blot with anti-MSH6 antibodies. MSH6-depleted U2OS cells were transfected with GFP-Polκ or GFP-REV1. 40 h later, the cells were irradiated with 15 J/m^2 UVC and further incubated for 12 h. The proportion of GFP-Polκ **(B)** or GFP-REV1 **(C)** expressing cells with more than 30 foci was determined.

Fig. S4. Depletion of MSH2 impairs post-UV PCNA-mUb in HCT116 cells. HCT116 cells were transfected with siMSH2 or siNC and exposed to UVC (15 J/m^2) radiation 72 h later. 4 h later, the triton-insoluble fractions were harvested and separated by SDS-PAGE. The levels of PCNA-mUb and MSH2 were detected by western blot with antibodies against PCNA and MSH2. The levels of Ku 80 were used as loading controls.

Fig. S5. MSH2 is required for optimal Rad18 foci formation after UV exposure. U2OS cells were transfected with siMSH2 or siNC and mock- **(A)** or UVC (15 J/m^2) irradiated **(B)** at 72 h following transfection. 4 h later, the cells were treated with 0.5% Triton X-100 for 5 min before fixation with 4% PFA to remove the soluble Rad18. The cells were then immunostained for Rad18 (red) in nuclei (DAPI, blue). Merge represents combined staining for Rad18 and for nuclei.

Fig. S6. Overexpression of MSH2 does not increase the levels of PCNA-mUb in 293T cells. 293T cells were transfected with Flag-MSH2 or Flag and exposed to UVC (15 J/m^2) radiation 40 h later. 4 h later, the triton-insoluble fractions were harvested and separated by SDS-PAGE. Levels of unmodified PCNA (PCNA) and mUb-PCNA were analyzed by western blot with anti-PCNA antibody and quantified using ImageJ. The levels of MSH2 were detected by western blot with antibodies against MSH2 or Flag.

Fig. S7. A MMR-deficient Flag-MSH2 (A834T) rescues REV1 focus formation in U2OS/ siMSH2 (3'-UTR) cells after UV irradiation. U2OS cells were transfected with siMSH2 (3'-UTR). Three days later, the cells were complemented with either wild-type (WT) or MMR-deficient Flag-MSH2 (A834T) and REV1 focus formation was measured as in **Fig. S2 (A)**.

Fig. S1, Lv *et al*

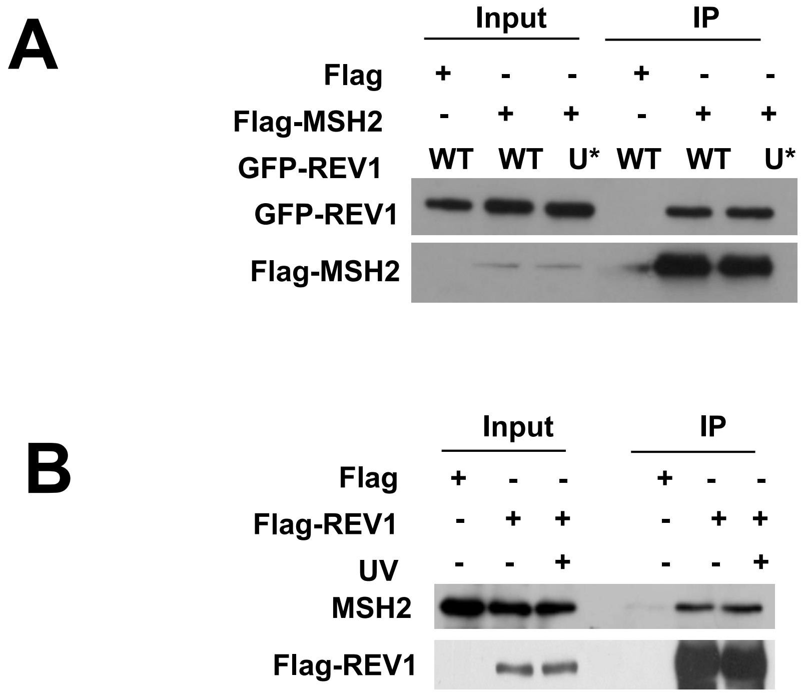

Fig. S2, Lv *et al*

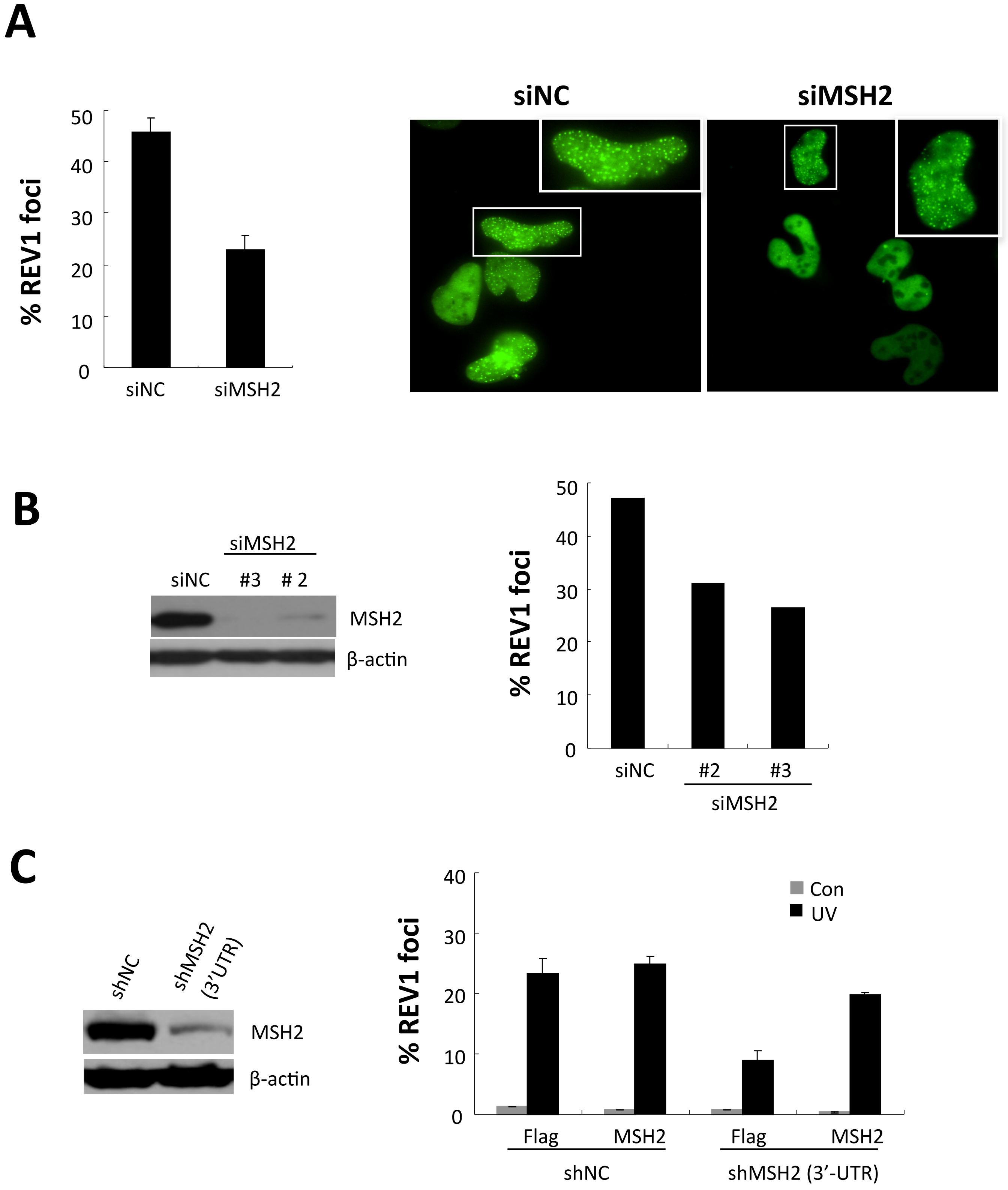

Fig. S3, Lv *et al*

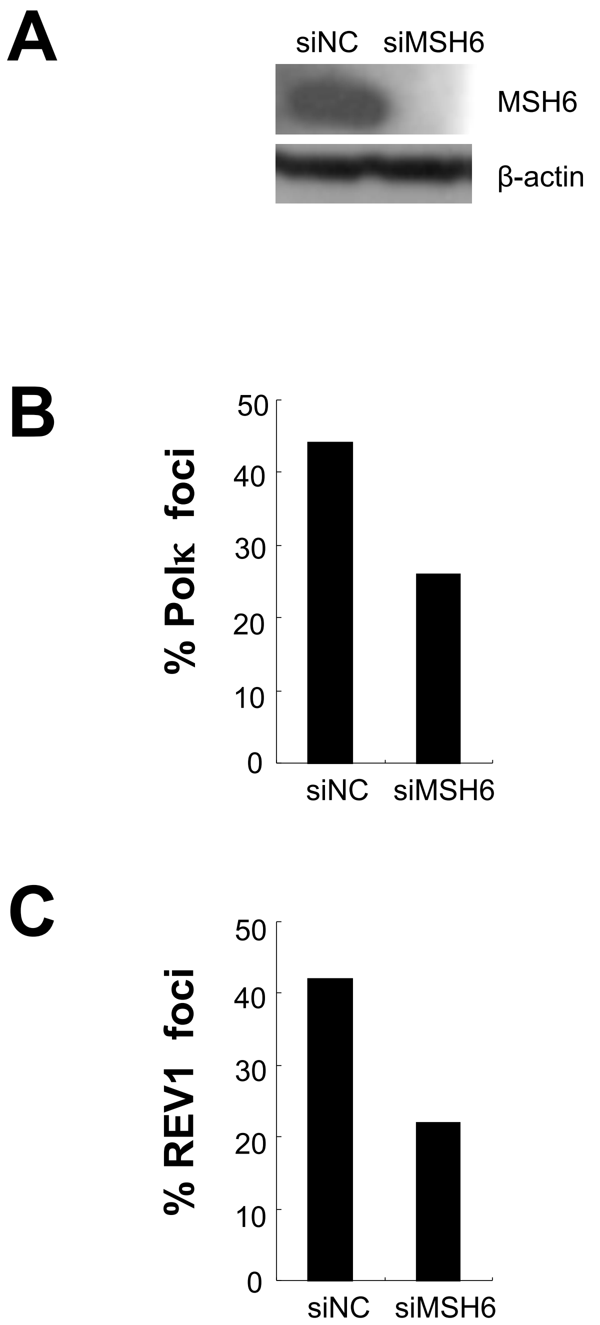

Fig. S4, Lv *et al*

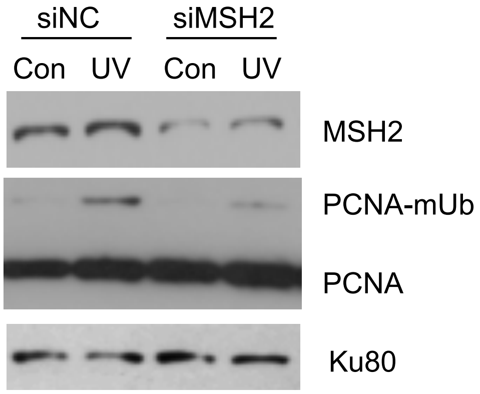

Fig. S5, Lv *et al*

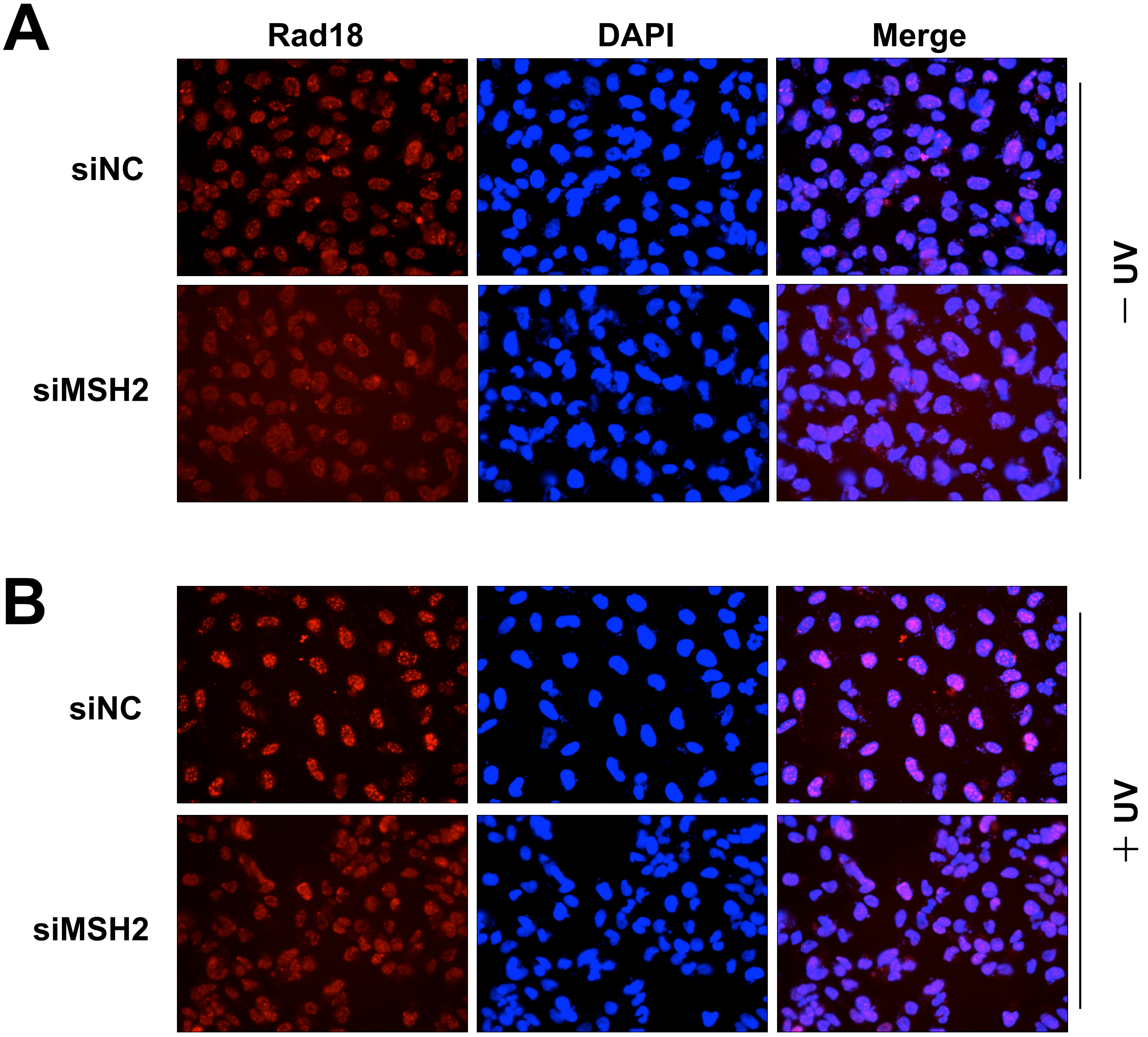

Fig. S6, Lv *et al*

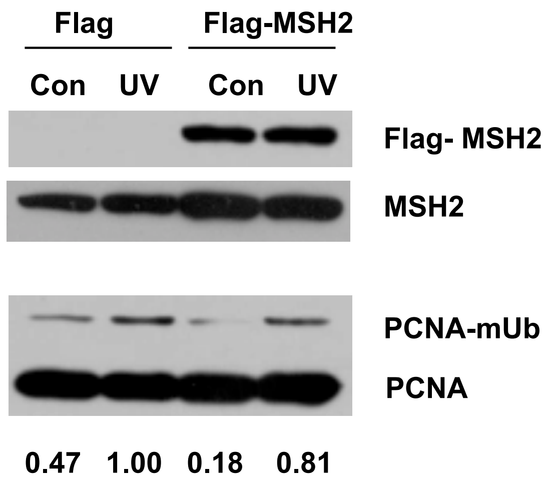

Fig. S7, Lv *et al*

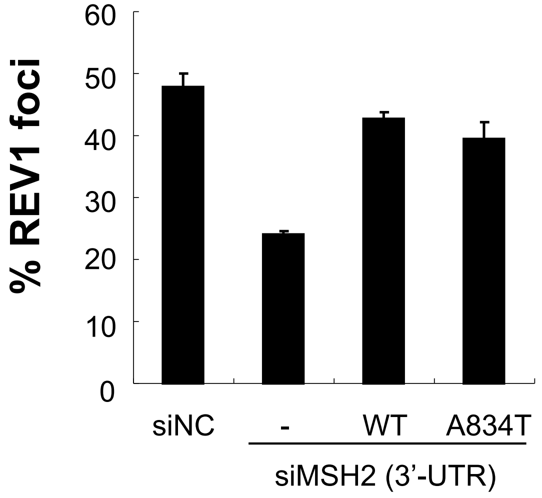

Supplement: Supplementary Data [file supp_gkt793_nar-01957-d-2013-File007.pdf]
